# Intermittent Fasting Resolves Dyslipidemia and Atherogenesis in Apolipoprotein E-Deficient Mice in a Diet-Dependent Manner, Irrespective of Sex

**DOI:** 10.3390/cells12040533

**Published:** 2023-02-07

**Authors:** Jules Mérian, Lamia Ghezali, Charlotte Trenteseaux, Thibaut Duparc, Diane Beuzelin, Vanessa Bouguetoch, Guillaume Combes, Nabil Sioufi, Laurent O. Martinez, Souad Najib

**Affiliations:** 1 Institut des Maladies Métaboliques et Cardiovasculaires, I2MC, Université de Toulouse, Inserm, Université Toulouse III—Paul Sabatier (UPS), UMR1297, 31432 Toulouse, France; 2 Lifesearch SAS, 195 Route d’Espagne, 31100 Toulouse, France

**Keywords:** intermittent fasting, glucose tolerance, hepatic steatosis, adipose tissue, reverse cholesterol transport, atherosclerosis

## Abstract

In humans and animal models, intermittent fasting (IF) interventions promote body weight loss, improve metabolic health, and are thought to lower cardiovascular disease risk. However, there is a paucity of reports on the relevance of such nutritional interventions in the context of dyslipidemia and atherosclerotic cardiovascular diseases. The present study assessed the metabolic and atheroprotective effects of intermittent fasting intervention (IF) in atherosclerosis-prone apolipoprotein E-deficient (*Apoe^-/-^*) mice. Groups of male and female *Apoe*^-/-^ mice were fed a regular (chow) or atherogenic (high-fat, high-cholesterol, HFCD) diet for 4 months, either *ad libitum* or in an alternate-day fasting manner. The results show that IF intervention improved glucose and lipid metabolism independently of sex. However, IF only decreased body weight gain in males fed chow diet and differentially modulated adipose tissue parameters and liver steatosis in a diet composition-dependent manner. Finally, IF prevented spontaneous aortic atherosclerotic lesion formation in mice fed chow diet, irrespective of sex, but failed to reduce HFCD-diet-induced atherosclerosis. Overall, the current work indicates that IF interventions can efficiently improve glucose homeostasis and treat atherogenic dyslipidemia, but a degree of caution is warranted with regard to the individual sex and the composition of the dietary regimen.

## 1. Introduction

Dietary interventions are broadly considered to be powerful non-pharmacological approaches not only for losing weight and adiposity but also for the prevention of metabolic and cardiovascular diseases in diverse model organisms [1,2,3,4].

Intermittent fasting (IF) has been promoted as an alternative dietary weight loss strategy to caloric restriction diets [5]. The IF regimen encompasses several patterns, including every-other-day fasting (feeding no calories on fasting days), every-other-day modified fasting (feeding less than 25% of caloric demand on fasting days), cyclic fasting (fasting on two to three days per week), and time-restricted fasting (limiting the interval of daily food intake to specific daytimes).

Accumulating evidence from both human and animal studies suggests that IF, in particular every-other-day fasting, has ample potential to improve cardiometabolic health [6,7,8,9,10].

Human studies have reported that IF interventions favor body weight loss. However, these studies have been primarily conducted in small populations including non-obese healthy individuals or metabolically healthy overweight or obese patients [11,12,13,14]. Moreover, Wilkinson et al. recently reported that IF interventions in humans efficiently promote body weight loss but only slightly improve glucose metabolism [15]. However, IF significantly reduced several cardiovascular risk factors, such as blood pressure and plasma atherogenic lipids levels, including triglycerides (TG) and low-density lipoprotein cholesterol (LDL-C) in patients with metabolic syndrome [15,16]. However, the dietary regimen compositions were poorly documented in these studies, and no study to date has assessed the effect of IF on patients with mild or advanced atherosclerosis.

In mice, IF intervention extends lifespan independently of sex and calorie intake [17]. In both sexes, IF protects mice from diet-induced obesity, glucose intolerance, insulin resistance, liver steatosis, systemic inflammation, and dyslipidemia [18,19]. These beneficial effects of IF on metabolic syndrome are mediated by diverse mechanisms including modulation of adipokine production [20], improvement in insulin sensitivity, reduction in oxidative stress and inflammation, and enhancement of adaptive stress responses such as autophagy, proteostasis, and endogenous antioxidant systems [17,21].

Three studies have analyzed the impact of IF in atherogenesis in atherosclerosis mouse models, apolipoprotein E- and low-density lipoprotein receptor-deficient mice (*Apoe*^-/-^ and *LDLr^-/-^*, respectively) [22,23,24], but they included only males, and the differences in the diet compositions and IF regimen patterns used in these studies have yielded contradictory results regarding glucose homeostasis and atherogenesis. 

In this context, the present study aimed to examine whether an IF intervention, in the form of an alternate-day fasting regimen, improves metabolic health and prevents atherogenesis in male and female *Apoe^-/-^* mice fed chow or atherogenic diet by analyzing lesions at early and advanced stages of atherosclerosis, respectively [25].

## 2. Materials and Methods

### 2.1. Ethics

All mouse experiments were approved by the institutional animal care and use committee CEEA122, under number 2019/022111492658, and were performed according to Inserm guidelines for the care and use of laboratory animals. All efforts were made to minimize animal suffering and the number of animals used.

### 2.2. Animals, Diets, and Feeding Interventions

**Figure 1:**
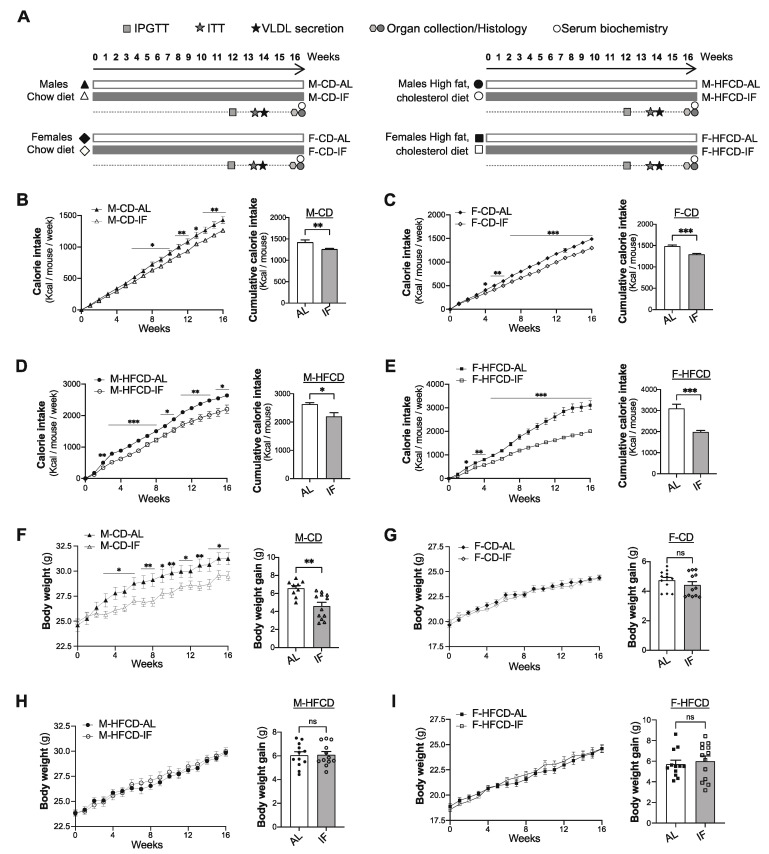
Intermittent fasting reduces body weight gain in *Apoe^-/-^* male mice fed chow diet. (**A**), Schematic outline of the experimental design: male or female *Apoe^-/-^* mice were divided into four groups that were assigned standard chow diet *ad libitum* (CD-AL group), intermittent fasting treatment of chow diet, by fasting every other day (CD-IF group) for 16 weeks, high-fat high-cholesterol diet *ad libitum* (HFCD-AL group), or intermittent fasting of HFCD (HFCD-IF group) for 16 weeks. Metabolic tests were performed at the indicated time, and blood and organ samples were collected from euthanized mice at the end of the treatment. (**B**–**E**) The calorie intake per mouse was calculated from the weekly calorie intake values per cage divided by the number of co-housed mice and expressed per week (left panel) and cumulatively over the 16 weeks of follow-up (right panel). Calorie intake in CD-fed males (**B**), CD-fed females (**C**), HFCD-fed males (**D**), and HFCD-fed females, *n* = 12–14 per group. (**F**–**I**) Change in body weight per week (left panel) and body weight gain after 16 weeks of treatment (right panel) in CD-fed males (**F**), CD-fed females (**G**), HFCD-fed males (**H**), and HFCD-fed females (**I**), *n* = 12–14 per group. Values are expressed as means ± SEM; ns: not significant; * *p* < 0.05, ** *p* < 0.01, *** *p* < 0.001. IPGTT: intraperitoneal glucose tolerance test, ITT: insulin tolerance test, VLDL: very low-density lipoprotein.

Apolipoprotein E-deficient (*Apoe^-/-^)* mice on a C57BL/6J background were supplied by the Jackson Laboratory (Bar Harbor, ME, USA). Animals were housed at 22 ± 1 °C on a 12 h light–dark cycle and had *ad libitum* access to water and a standard normal chow diet (CD, 67% carbohydrates, 9% fat, V1534-000, ssniff^®^, Soest, Germany). At 6 weeks of age, male and female mice were randomized into experimental groups based on body weight (RandoMice software, Leiden, the Netherlands [26]) and co-housed (3–4 mice per cage) for 2 weeks to allow for acclimation prior to feeding intervention for 16 weeks. The experimental groups consisted of (1) *ad libitum* feeding with CD (CD-AL group), (2) *ad libitum* feeding with CD in alternation to fasting every 24 h [24,27], namely intermittent fasting (CD-IF group), (3) *ad libitum* feeding with a high-fat high-cholesterol diet (HFCD, 50% carbohydrates, 20% fat with 16% of cocoa butter, and 1.25% cholesterol, SAFE A04, SAFE, Augy, France) (HFCD-AL group), and (4) *ad libitum* feeding with HFCD in alternation with fasting every 24 h (HFCD-IF group). Food intake (kcal) and body weight (g) were monitored weekly, and the metabolic efficiency was calculated as g of body weight gained per millijoule (MJ) of food consumed during the 16 weeks of intervention. The mice were then euthanized, and blood, adipose tissues, liver, heart, and aorta were collected for biochemical and histological analysis. Organs and tissues were frozen for further analysis. The feeding intervention protocol (Figure 1A) was repeated in three independent cohorts.

### 2.3. Plasma Lipid Analysis

Plasma lipids were assessed as previously described [28]. Briefly, the concentrations of plasma triglycerides (TG), high-density lipoprotein cholesterol (HDL-C), total cholesterol, and non-esterified fatty acids were determined using commercial colorimetric kits (Biolabo SAS, Maizy, France) based on GPO and CHOD-PAP detection methods, coupling enzymatic reaction, and detection of reaction end products by spectrophotometry (*CLARIOstar Plus* microplate reader, BMG Labtech, Ortenberg, Germany).

### 2.4. Glucose and Insulin Tolerance Test

An intraperitoneal (i.p.) glucose tolerance test (IPGTT) was performed at 12 weeks of feeding intervention. Animals were fasted overnight before the experiments. Mice were administered glucose as a single bolus i.p. injection at 1 g/kg of body weight. Blood samples for plasma glucose measurement were collected from the tail vein 30 min before and 30, 60, 90, and 120 min after glucose administration. Glucose was measured using an Accu-Chek^®^ glucometer (Roche Diabetes Care, Meylan, France). The plasma (5 µL sample) insulin concentration was determined 30 min before and 15 min and 60 min after glucose loading using an ultrasensitive ELISA kit (#10-1247, Mercodia AB, Uppsala, Sweden).

An insulin tolerance test (ITT) was performed at 14 weeks of feeding intervention in the same mouse groups that were tested for IPGTT. Animals were fasted 5 h before the experiment and received a single i.p. bolus injection of insulin at 0.5 U/kg of body weight. Blood was collected from the tail vein 30 min before and 15, 30, 60, 90, and 120 min after insulin administration. Glucose was measured using the Accu-Chek^®^ glucometer (Roche Diabetes Care, Meylan, France).

### 2.5. Hematoxylin and Eosin (H&E) Staining

Tissue histology was performed as previously described [28]. Briefly, liver, subcutaneous white adipose tissue (SC-WAT), and brown adipose tissue (BAT) were fixed in 4% paraformaldehyde for 24 h at room temperature, dehydrated, and embedded into paraffin. The tissues were sectioned into 5 μm slices. The sections were incubated for 30 min in Mayer’s hematoxylin solution, rinsed with distilled water for 5 min, and then incubated in saturated lithium carbonate solution for 15 s. The sections were then rinsed again with distilled water for 3 min and finally placed in 0.5% alcoholic eosin solution for 30 s. The slides were scanned with a NanoZoomer scanner 2.0 RS (Hamamatsu, Hamamatsu city, Japan) at 40× magnification. The images shown are representative results of at least three biological replicates.

### 2.6. RNA Extraction and Quantitative Real-Time PCR Analysis

RNA extraction and quantitative real-time PCR were performed as previously described [28]. Briefly, total RNA was prepared from liver or adipose tissues using QIAzol lysis reagent (QIAGEN, Germantown, MD, USA). The concentration of extracted RNAs of each sample was determined using a NanoDrop™ spectrophotometer (Thermo Fisher Scientific, Waltham, MA, USA). Reverse transcription of mRNA (1 μg) was performed using M-MLV Reverse Transcriptase (Promega, Madison, WI, USA) in the presence of a random primer (oligo(dT); 2 μL, 500 μg/mL; Promega) and 0.5 μL RNaseOUT™ (Thermo Fisher Scientific, Waltham, MA, USA). After incubation for 8 min at 75 °C to inactivate DNase, 200 U of M-MLV reverse transcriptase was added with subsequent incubation at 25 °C for 10 min and then at 37 °C for 1 h. The reverse transcription reaction was terminated by heating at 95 °C for 5 min and then chilling and storing at −80 °C.

Real-time quantitative PCR was performed using the SsoFast™ EvaGreen^®^ Supermix (Bio-Rad, Marnes-la-Coquette, France). PCR was carried out in a 96-well format with 20 μL reactions containing SsoFast™ EvaGreen^®^ Supermix (10 μL), cDNA (2 μL), gene-specific primers (0.5 μL), and DNase/RNase-free water (7 μL). To perform RT-PCR analyses, the relative gene expression of each transcript was normalized to the reference gene *Gapdh* by the 2^−∆∆Ct^ method and further normalized to the relative expression of the respective controls (i.e., *ad libitum*-fed group mice). The oligonucleotide sequences are provided in the Supplementary data section, Table S1.

### 2.7. Liver Lipid Content

The concentration of lipids in the liver was determined as previously described [28], according to Folch et al. [29]. Briefly, frozen liver tissue was homogenized in phosphate buffer (pH 7.4) until complete tissue lysis. Lipids were extracted by mixing the liver lysate with a 2:1 chloroform-to-methanol solution. After centrifugation, the chloroform phase was evaporated under nitrogen, and the dried residue was solubilized in isopropanol. Triglycerides and cholesterol were measured using commercial kits based on the CHOD-PAP and GPO-PAP detection methods (Biolabo SAS, Maizy, France). The results are expressed as micrograms of lipid per gram of liver.

### 2.8. Liver Reactive Oxygen Species and TBARS

Reactive oxygen species (ROS) in liver were assayed as described [28]. Briefly, liver tissue was homogenized in a cold buffer (150 mM KCl, 20 mM Tris base, 0.5 mM EGTA, 1 mM MgCl_2_, 5 mM glucose, and 0.5 mM octanoic acid at pH 7.4). The ROS-reactive fluorescent probe H_2_-DCFDA (Molecular Probes) was then added to the liver lysate and incubated at 37 °C for 30 min. The reaction was stopped with 0.1 M HCl in 70% of ethanol. The samples were centrifuged, and the supernatants were collected and neutralized by the addition of 1 M NaHCO_3_ solution. The fluorescence was determined (485 nm excitation, 535 nm emission) using a *CLARIOstar Plus* microplate reader (BMG Labtech, Ortenberg, Germany). The relative fluorescence units were normalized to the liver tissue weight used for each sample measurement, and the results are expressed as the percentage of fluorescence of the IF groups versus the AL groups. Lipid peroxidation was assessed as described [28] by measuring the concentration of thiobarbituric acid response substrates (TBARS), which are by-products of lipid peroxidation. Liver homogenate was incubated with 200 μL of 0.6% TBA and 10 μL of 1% H_3_PO_4_ solutions at 95 °C for 1 h. After the addition of 200 μL butanol, the colored phase was extracted. Fluorescence was measured (515 nm excitation, 548 nm emission) using a *CLARIOstar Plus* microplate reader (BMG Labtech, Ortenberg, Germany). The TBARS concentration was extrapolated from a standard curve. The results were normalized to the liver tissue weight used for each sample measurement and are expressed as the percentage of TBARS in the liver of the IF groups versus the AL groups.

### 2.9. Cannulation of the Common Bile Duct and Collection of Hepatic Bile

At the end of the IF intervention, a cohort of 6–7 mice per group was anesthetized by intraperitoneal injection of ketamine/xylazine. The common bile duct was ligated close to the duodenum, and then the gallbladder was punctured and cannulated with a polyethylene-10 catheter. After 30 min, newly secreted bile was collected for 30 min by gallbladder cannulation. During bile collection, the body temperature was stabilized using a temperature mattress. Bile flow (μL/min/100 g of body weight) was determined gravimetrically assuming a density of 1 g/mL for bile.

### 2.10. Biliary Lipid Analyses

For bile acid analysis [30], 1 μL of bile sample was diluted with 99 μL of Milli-Q^®^ water and then incubated with the reagent solution (6 mg NAD, 0.5 M hydrazine hydrate buffer, 0.05 M Na-pyrophosphate) for 4 min. The mix was then incubated with the solution (0.03 M Tris-EDTA; 0.3 U/mL 3-alpha-OH steroid dehydrogenase), and the bile acid concentration was determined by spectrophotometry (excitation of 340/330 nm/emission of 440/420 nm, *CLARIOstar Plus* microplate reader, BMG Labtech, Ortenberg, Germany). For phospholipid (PL) analysis, 1 μL of bile sample was diluted with 49 μL of Milli-Q^®^ water and then incubated with the reagent solution (100 mM MOPS, pH 8; 0.55 mM HVA; 20 mM CaCl_2_; 11 U/mL phospholipase-D; 1.66 U/mL peroxidase; 0.1% Triton X-100) for 4 min. The mix was then incubated with a start reagent (1 M MOPS, pH 8, 50 U/mL choline oxidase), and the PL concentration was determined by spectrophotometry (excitation 340/330 nm/emission 440/420 nm using a *CLARIOstar Plus* microplate reader, BMG Labtech, Ortenberg, Germany). For cholesterol analysis, 1 μL of bile sample was diluted with 29 μL of Milli-Q^®^ water and then incubated with the reagent solution (100 mM MOPS, pH 8, 0.25 mM HVA; 0.1% Triton X-100) for 4 min. The mix was then incubated with a start reagent (0.1 M MOPS, pH 8, 0.06 U/mL cholesterol oxidase, 0.15 U/mL cholesterol esterase, 0.45 U/mL peroxidase, 0.06 mM taurocholate), and the cholesterol concentration was determined by spectrophotometry (excitation of 330/340 nm/emission of 420/440 nm, *CLARIOstar Plus* microplate reader, BMG Labtech, Ortenberg, Germany).

### 2.11. Atherosclerotic Lesion Analysis

Atherosclerosis progression was measured in euthanized mice at the end of the intervention, as previously described [31] (*n* = 5–6 per group). Briefly, the heart linked to the ascending aorta was carefully removed and snap-frozen at −80 °C. Peripheral fat was removed from defrosted heart samples, under a binocular magnifier, and samples were embedded in OCT and frozen at −80 °C for serial 10 μm-thick cryosectioning. Serial cross-sections of three valve leaflets from the aortic root were sectioned (five sections per slide, from 200 to 800 μm from the base of the heart to the aortic arch). The slides were stained with Oil Red O (ORO) to visualize neutral lipids and counterstained with hematoxylin. Images of all stained slides were captured with a NanoZoomer scanner 2.0 RS (Hamamatsu, Hamamatsu city, Japan). The areas of lesions were quantified using ImageJ software for image analysis and represented as the average of the lipid-stained areas for each distance from the heart [31].

### 2.12. Statistical Analysis

All analyses of the results were performed using GraphPad prism V 6.06 software. The data are expressed as means ± the standard error of the mean (SEM). Significance was determined by the Mann–Whitney non-parametric test for comparison between two groups, intermittent fasting vs. *ad libitum.* A *p*-value of less than 0.05 was considered significant.

## 3. Results

### 3.1. Food Intake and Body Weight Gain

Two-month-old male (M) and female (F) *Apoe*^-/-^ mice were randomly assigned to one of the four experimental groups (Figure 1A): *ad libitum* feeding with chow diet (CD-AL), intermittent feeding with CD (CD-IF), *ad libitum* feeding with high-fat high-cholesterol diet (HFCD-AL), and intermittent feeding with HFCD (HFCD-IF). As expected, for all sexes and diet compositions, the cumulative calorie intake was significantly lower in the IF groups compared with the AL groups (Figure 1B–E). Strikingly, despite this reduction in cumulative calorie intake in all IF groups, the intermittent fasting intervention only decreased body weight gain in males fed CD (Figure 1F) while no effect of IF on body weight gain was observed in males fed HFCD (Figure 1H) or in females fed CD or HFCD (Figure 1G–I). Metabolic efficiency, which reflects metabolic control of weight gain measured as the part of ingested energy that is stored as extra body energy deposits [32], was lower in M-CD-IF compared to M-CD-AL. This effect of IF was not observed in females fed CD (Supplementary Figure S1A) or in males fed HFCD (Supplementary Figure S1B, left panel). However, in females fed HFCD, IF led to an increase in metabolic efficiency (Supplementary Figure S1B, right panel). 

These data indicate that IF regulates body weight gain and metabolic efficiency in *Apoe*^-/-^ mice in a sex- and diet composition-dependent manner.

### 3.2. Lipid and Glucose Homeostasis

**Figure 2:**
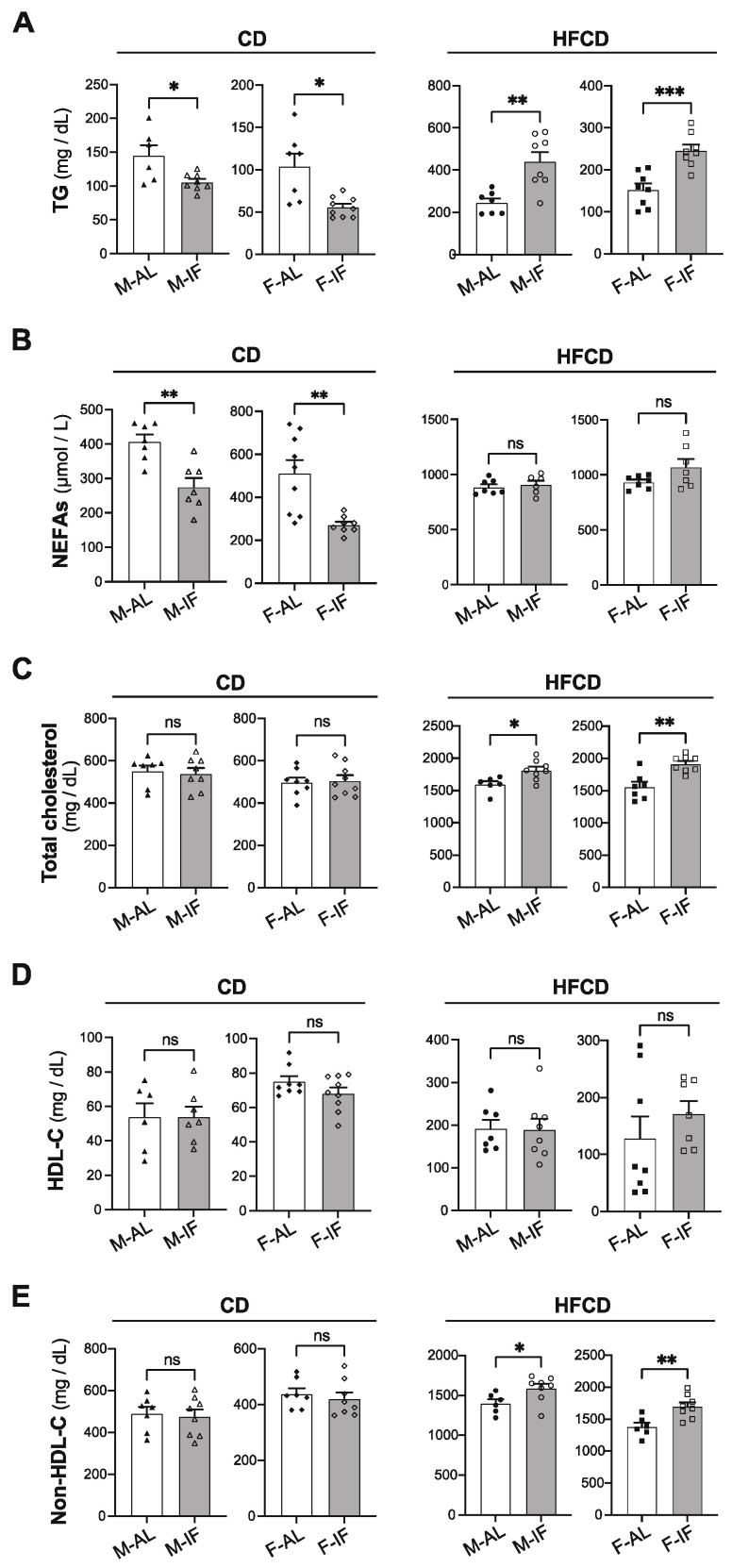
Intermittent fasting reduces hypertriglyceridemia in *Apoe*^-/*-*^ mice fed CD, while it exacerbates HFCD-induced dyslipidemia. Plasma levels of TG (**A**), NEFAs (**B**), total cholesterol (**C**), HDL-C (**D**) and non-HDL-C (**E**) in *ad libitum* (AL) or intermittent fasting (IF) *Apoe*^-/-^ mice fed chow diet (CD) or high-fat high-cholesterol diet (HFCD). Values are expressed as means ± SEM (*n* = 6–9 per group); ns; not significant, * *p* < 0.05, ** *p* < 0.01, *** *p* < 0.001. HDL-C, high-density lipoprotein cholesterol; TG, triglycerides; NEFAs, non-esterified fatty acids.

Compared to the AL control group, IF intervention in *Apoe^-/-^ mice* fed CD significantly lowered the plasma levels of TG and NEFA in both sexes, while this condition did not affect total cholesterol, *HDL-C* and non-HDL-C levels (Figure 2A–E, left panels). Conversely, when *Apoe*^-/-^ mice were fed HFCD, IF intervention favored a dyslipidemic profile in both sexes, characterized by higher levels of plasma TG, total cholesterol, and non-HDL-C and similar levels of HDL-C and FFA (Figure 2A–E, right panel).

**Figure 3:**
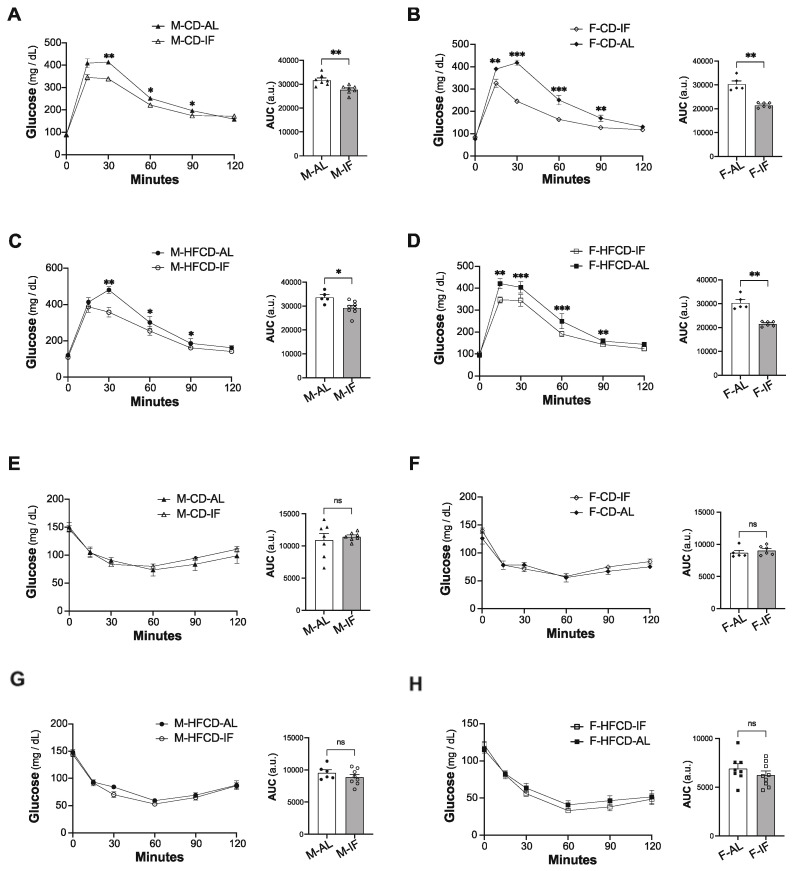
Intermittent fasting in *Apoe*^-/*-*^ mice improves glucose tolerance. (**A**–**D**) Intraperitoneal glucose tolerance test (IPGTT). At 12 weeks of intervention, *ad libitum* (AL) and intermittent fasting (IF) *Apoe*^-/-^ mice were fasted for 16 h and received an intraperitoneal injection of glucose (1 g/kg). Glycemia was measured at the indicated times (left panel), and the respective area under the curve (AUC) was calculated (right panel) for CD-fed males (**A**), CD-fed females (**B**), HFCD-fed males (**C**), and HFCD-fed females (**D**). (**E**–**H**) Intraperitoneal insulin tolerance test (ITT). Two weeks after IPGTT, the same mice were fasted for 5 h and received an intraperitoneal injection of insulin (0.5 U/kg). Glycemia was measured at the indicated times (left panel), and the respective area under the curve (AUC) was calculated (right panel) for CD-fed males (**E**), CD-fed females (**F**), HFCD-fed males (**G**), and HFCD-fed females (**H**). Values are expressed as means ± SEM (*n* = 5–9 per group); ns: not significant; * *p* < 0.05, ** *p* < 0.01, *** *p* < 0.001.

Baseline fasting glycaemia did not differ between the IF and AL groups (Figure 3A–D). In comparison to AL feeding, IF intervention resulted in less increase and faster clearance of blood glucose upon glucose loading (IPGTT), in both sexes and with any diet composition (Figure 3A–D). This improvement in glucose tolerance mediated by IF was not due to changes in plasma insulin levels (Supplementary Figure S2). Accordingly, the insulin sensitivity was comparable between the IF groups and their respective AL control groups, irrespective of sex and diet composition (Figure 3E–H), confirming that IF improves glucose tolerance in an insulin-independent manner. 

Altogether, these results indicate that IF intervention improves glucose tolerance in *Apoe^-/-^* mice, independently of sex and diet composition, and partially resolves hypertriglyceridemia in mice fed CD, but it worsened dyslipidemia when mice were fed HFCD.

### 3.3. Adipose Tissue Features

**Figure 4:**
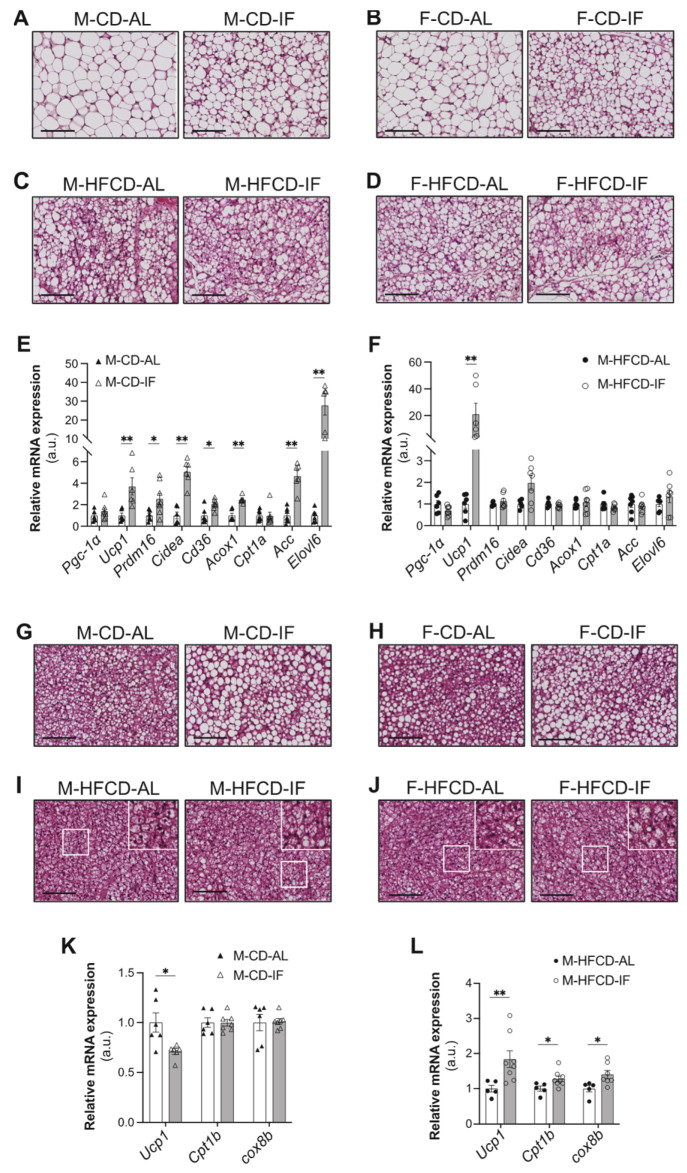
Intermittent fasting in *Apoe*^-/*-*^ mice regulates adipose tissue phenotypes depending on the diet. (**A**–**D**) Representative images of H&E staining of WAT tissue sections of *ad libitum* (AL) or intermittent fasting (IF) CD-fed males (**A**), CD-fed females (**B**), HFCD-fed males (**C**), and HFCD-fed females (**D**). Scale bar: 100 μm, *n* = 4 per group. (**E**,**F**) WAT expression of genes related to browning and lipid metabolism in *ad libitum* (AL) or intermittent fasting (IF) CD-fed males (**E**) and HFCD-fed males (**F**). (**G**–**J**) Representative images of H&E staining of BAT tissue sections of *ad libitum* (AL) or intermittent fasting (IF) CD-fed males (**G**), CD-fed females (**H**), HFCD-fed males (**I**), and HFCD-fed females (**J**). Scale bar: 100 μm, *n* = 3 per group. (**K**,**L**) BAT expression of genes related to BAT activation in *ad libitum* (AL) or intermittent fasting (IF) CD-fed males (**K**) and HFCD-fed males (**L**). Upper right insets (**I**,**J**): zoom-in on white squares. Values are expressed as means ± SEM (*n* = 6–7 per group); * *p* < 0.05, ** *p* < 0.01.

The effect of IF on weight loss might be consequent to WAT browning [33]. IF intervention in any group did not affect the subcutaneous adipose tissue (SC-AT) weight (Supplementary Figure S3A,B) but histological analysis of SC-AT sections showed that IF resulted in a profound morphological transformation towards a brown adipose tissue (BAT)-like phenotype, as numerous clusters of multilocular adipocytes were observed (Figure 4A,B). HFCD by itself (i.e., under AL feeding conditions) induced a transformation towards a BAT-like phenotype, as observed in Figure 4A–D (left panels), as reported in *Apoe^-/-^* mice fed a high-fat diet [34,35].

Given that IF favored a BAT-like phenotype independently of sex, we analyzed gene expression in male mice only. In support of the histological analysis, when mice were fed CD, IF induced a significant increase in the expression of browning genes in SC-AT (Figure 4E), including uncoupling protein 1 (*Ucp1*), PR domain containing 16 (*Prdm16*), and cell death-inducing DFFA-like effector A (*Cidea*). IF also induced a significant increase in lipolysis-related genes, including cluster of differentiation 36 (*Cd36*), acyl-CoA oxidase 1 (*Acox1*), and elongation of very long-chain fatty acids protein 6 (*Elovl6*), as well as acetyl-CoA carboxylase alpha (*Acaca*), a rate-limiting enzyme for fatty acid synthesis (Figure 4E). Interestingly, the *Ucp1* mRNA expression level was dramatically upregulated by IF in mice fed HFCD (Figure 4F), indicating that IF stimulates WAT browning on top of the HFCD effect. No difference was observed between the AL and IF groups in gene expression of peroxisome proliferator-activated receptor gamma coactivator 1-*alpha* (*Pgc1-α)* or carnitine palmitoyltransferase-1 (*Cpt1-α*), the rate-limiting enzyme of fatty acid oxidation (Figure 4E,F).

Together with AT browning, IF intervention increased the weight of BAT in mice of both sexes fed CD (Supplementary Figure S3C). A similar effect of IF was observed when mice were fed HFCD, but only in males (Supplementary Figure S3D). Intriguingly, brown fat cells from both male and female mice fed CD and undergoing IF intervention acquired a white-like unilocular adipocyte phenotype compared to their control AL mice (Figure 4G,H). However, under HFCD, no evidence of a difference in brown adipocyte morphology was observed between the AL and IF groups (Figure 4I,J). Consistent with our histological analysis, under CD, the mRNA level of *Ucp1* was significantly reduced in the BAT of male mice submitted to IF compared to their AL controls. However, the expressions of the other brown adipocyte marker genes, *CPT1-α* and cytochrome *c* oxidase subunit 8b (*Cox8b*), were not modified by IF (Figure 4K). Interestingly, under HFCD, IF induced a significant increase in the expression of all of these thermoregulatory genes (Figure 4L), suggesting that brown adipocytes are activated by IF when mice are subjected to HFCD. 

Together, our data demonstrate that in *Apoe^-/-^* mice fed CD, IF differentially regulates the WAT and BAT morphologies and features by reprogramming lipid metabolism and browning gene expression in WAT and by inducing a whitening program in BAT. However, when mice are under HFCD, IF induces both browning of WAT and activation of BAT.

### 3.4. Hepatic Lipid Metabolism

**Figure 5:**
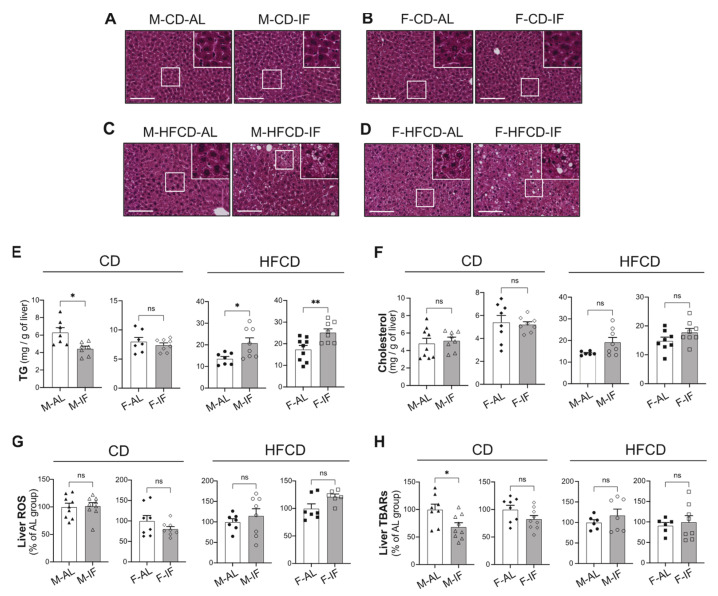
Intermittent fasting reduced hepatic triglyceride content in *Apoe^-/-^* male mice fed chow diet, while it exacerbated HFCD-induced steatosis. (**A**–**D**) Representative images of H&E staining of sections of liver tissue of *ad libitum* (AL) and intermittent fasting (IF) CD-fed males (**A**), CD-fed females (**B**), HFCD-fed males (**C**), and HFCD-fed females (**D**). Scale bars: 250 μm. (**E**) Liver triglyceride content in AL and IF CD-fed mice (left panel) and HFCD-fed mice (right panel). (**F**) Liver cholesterol content in AL and IF CD-fed mice (left panel) and HFCD-fed mice (right panel). (**G**) Liver reactive oxygen species (ROS) content in AL and IF CD-fed mice (left panel) and HFCD-fed mice (right panel). (**H**) Liver thiobarbituric acid response substrates (TBARS) level in AL and IF CD-fed mice (left panel) and HFCD-fed mice (right panel). Upper right insets (**A**–**D**): zoom-in on white squares. Values are expressed as means ± SEM (*n* = 6–9 per group); ns: not significant; * *p* < 0.05, ** *p* < 0.01.

The effect of IF on hepatic lipid deposition was first examined in H&E-stained liver sections. Under CD, the histological morphology of the liver was similar between the AL and IF groups, with a normal structure of the liver cells in both groups in a sex-independent manner (Figure 5A,B). However, we found that IF significantly reduced hepatic triglyceride (TG) levels in males fed CD but not in females (Figure 5E, left panel). In contrast, in mice fed HFCD, the abundance and size of lipid droplets were higher in the IF groups than in the AL groups (Figure 5C,D). Accordingly, IF in mice fed HFCD was associated with increased levels of hepatic TG, independently of sex (Figure 5E, right panel). The hepatic cholesterol content and ROS levels, documenting oxidative stress, were similar between the IF groups and their respective AL control groups (Figure 5F,G), and the ability of IF to reduce liver TBARS, which are by-products of lipid peroxidation, was strictly limited to males fed CD (Figure 5H). 

**Figure 6:**
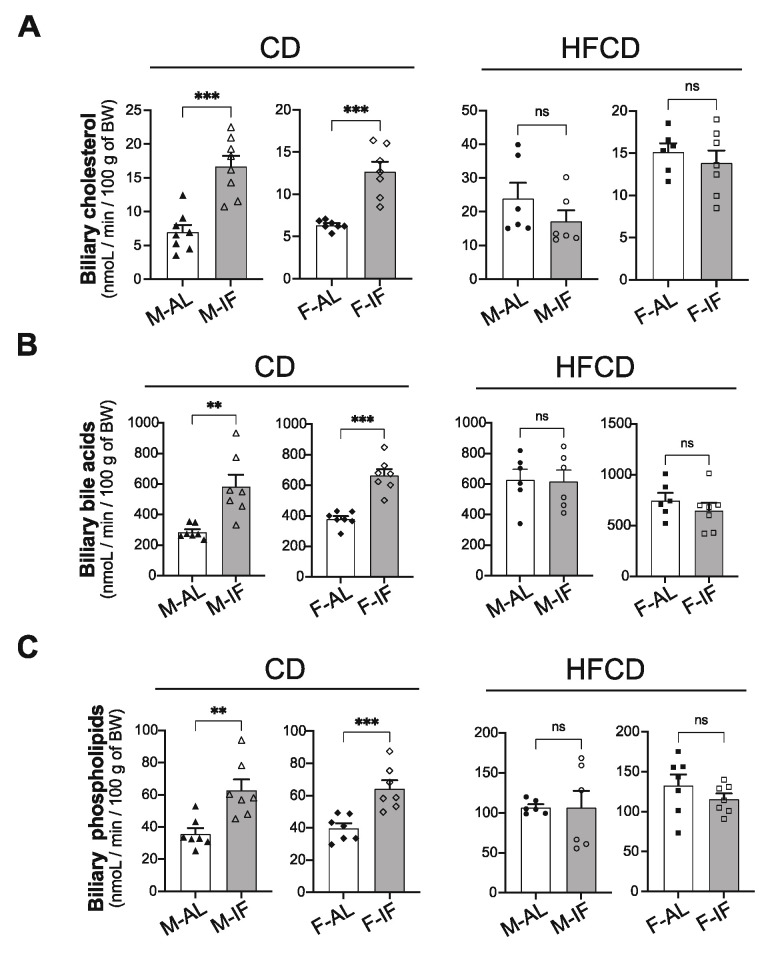
Intermittent fasting increased biliary lipid secretion in *Apoe^-/-^* mice fed CD. The gallbladder was cannulated, and bile was collected for 30 min, after a stabilization time of 30 min. Biliary secretions of cholesterol (**A**), bile acids (**B**) and phospholipids (**C**) were determined in *ad libitum* (AL) or intermittent fasting (IF) *Apoe*^-/-^ mice fed chow-diet (CD) or high-fat high-cholesterol diet (HFCD). Values are expressed as means ± SEM (*n* = 6–7 per group); ns, not significant; ** *p* < 0.01, *** *p* < 0.001.

We then examined hepatic lipid catabolism and fluxes. First, the rate of hepatic VLDL-TG secretion was reduced in all IF groups compared with the AL groups (Supplementary Figure S4A–D), indicating that IF efficiently lowered TG secretion by the liver, independently of the diet composition. We then examined hepatic biliary lipid secretion, which is considered to be the last step of the reverse cholesterol transport pathway by which the body removes excess cholesterol [36]. In mice fed CD, IF intervention significantly increased the biliary secretion of cholesterol, bile acids and phospholipids, irrespective of sex (Figure 6A–C, left panels). Of note, the effect of IF on the biliary lipid secretion was lost when mice were fed HFCD (Figure 6A–C, right panels).

**Figure 7:**
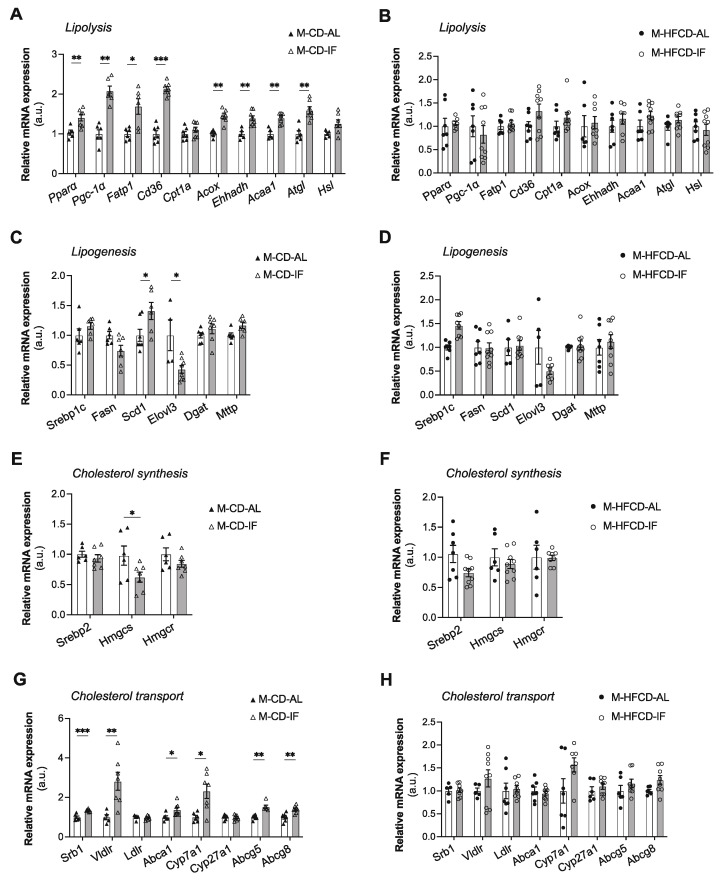
Intermittent fasting induced significant changes in the hepatic expression of genes involved in lipid and cholesterol metabolism only in males fed CD. (**A**,**B**) Expression of genes related to lipolysis in *ad libitum* (AL) or intermittent fasting (IF) CD-fed males (**A**) and HFCD-fed males (**B**). (**C**,**D**) Expression of genes related to lipogenesis in *ad libitum* (AL) or intermittent fasting (IF) CD-fed males (**C**) and HFCD-fed males (**D**). (**E**,**F**) Expression of genes related to cholesterol synthesis in *ad libitum* (AL) or intermittent fasting (IF) CD-fed males (**E**) and HFCD-fed males (**F**). (**G**,**H**) Expression of genes related to cholesterol transport in *ad libitum* (AL) or intermittent fasting (IF) CD-fed males (**G**) and HFCD-fed males (**H**). Values are expressed as means ± SEM (*n* = 5–8 per group); * *p* < 0.05, ** *p* < 0.01, *** *p* < 0.001.

We then evaluated the expression of genes involved in lipolysis, fatty acid and cholesterol synthesis, and cholesterol transport in the liver of *Apoe^-/-^* male mice fed CD and HFCD. In CD-fed mice, IF increased the hepatic expression of genes involved in lipid catabolism, including the transcriptional factor peroxisome-proliferator-activated-receptor-α (*Ppar-α)* and its co-activator *Pgc1-α*, as well as genes involved in the uptake and oxidation of fatty acids such as fatty acid transport protein 1 (*Fatp1*), *Cd36*, acyl-CoA oxidase 1 (*Acox1*), L-bifunctional protein (*Ehhadh*), and acetyl-CoA acyltransferase 1 (*Acaa1*). Interestingly, gene expression of adipose triglyceride lipase (*Atgl*) and hormone-sensitive lipase (*Hsl*), which are key enzymes involved in intracellular degradation of triacylglycerols, were also increased in the IF groups compared with the AL groups when mice were fed CD (Figure 7A). However, when mice were fed HFCD, IF had no effect on the expression of any of the above-mentioned genes (Figure 7B). We then explored the expression of lipogenic genes. Intriguingly, we found that under CD, the mRNA expression level of stearoyl-coenzyme A desaturase 1 (*Scd1*), the rate-limiting enzyme involved in the synthesis of monounsaturated fatty acids, was increased in the IF groups compared to the AL groups (Figure 7C). This increase could also explain the decrease in the liver TG content, as SCD1 has been reported to promote the catabolism of TG by its product, oleic acid, which upregulates ATGL and HSL [37]. We also found that the mRNA expression level of fatty acid elongase, *Elovl3*, which is involved in the synthesis of saturated and monounsaturated very long-chain fatty acids, was reduced (Figure 7C). IF had no effect on the mRNA expression level of the other lipogenic genes examined, including the transcription factor controlling fatty acid biosynthesis, sterol regulatory element-binding protein-1c (*Srebp-1c*), fatty acid synthase (*Fasn*), acyl CoA:diacylglycerol acyltransferase 2 (*Dgat2*), and microsomal triglyceride transfer protein (*Mttp*) (Figure 7C). Intriguingly, under HFCD, IF significantly increased the mRNA level of *Srebp-1c* only, without affecting the expression of its target genes *Fasn* and *Scd1*, or the expression of *Elovl3*, *Dgat*, and *Mttp* (Figure 7D).

The expression of cholesterol metabolism-related genes was also examined. We found that when mice were under CD, IF did not affect the mRNA level of sterol regulatory element-binding protein-2 (*Srebp-2*), a transcription factor that activates genes involved in cholesterol biosynthesis, nor did it affect the mRNA level of its target gene HMG-CoA reductase (*Hmgcr*), while it significantly decreased HMG-CoA synthase 1 (*Hmgs1*) gene expression (Figure 7E). Interestingly, IF also upregulated gene expression of the scavenger receptor class B type 1 (*Srb-1*) and very low-density lipoprotein receptor (*Vldlr*), involved in hepatic cholesterol uptake, the ATP-binding cassette transporter A1 (*Abca1*), involved in HDL cholesterol efflux and reverse cholesterol transport (RCT), and the cholesterol 7 alpha-hydroxylase A1 (*Cyp7a1*), and ATP-binding cassette G5 (*Abcg5*) and G8 (*Abcg8*) involved in cholesterol excretion (Figure 7G). When mice were fed HFCD, IF failed to modulate the expression level of genes involved in cholesterol biosynthesis and transport (Figure 7F,H).

Altogether, these results indicate that IF intervention in *Apoe^-/-^* mice fed CD triggers liver transcriptional reprogramming of lipid metabolism genes, which is associated with lower hepatic storage and secretion of TG and higher biliary cholesterol secretion, regardless of the sex (except for TG storage). Most of these beneficial effects of IF intervention on lipid metabolism in the liver were lost when mice were fed HFCD, while hepatic steatosis was worsened.

### 3.5. Atherosclerosis

**Figure 8:**
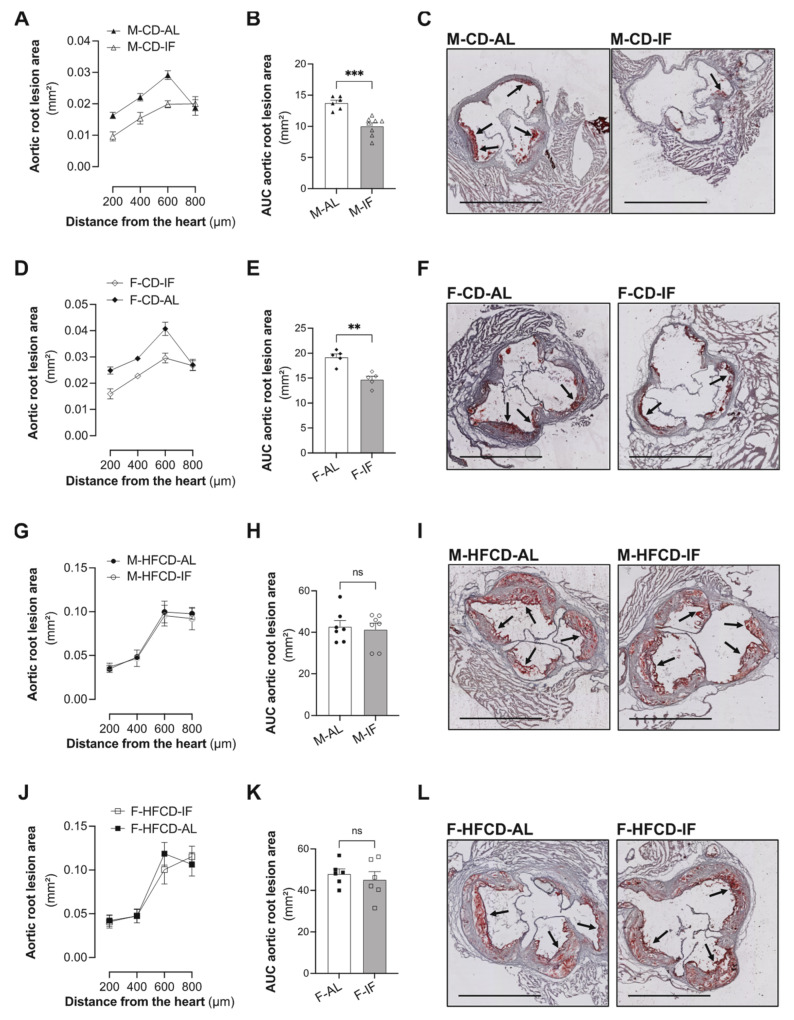
Intermittent fasting reduced atherosclerotic lesions only when *Apoe^-/-^* mice were fed CD. (**A**,**D**,**G**,**J**) Quantification of the Oil Red O-stained aortic root at the indicated distances from the heart of *ad libitum* (AL) or intermittent fasting (IF) males fed CD (**A**), females fed CD (**D**), males fed HFCD (**G**), and females fed HFCD (**J**). (**B**,**E**,**H**,**K**) Calculation of the AUC regarding the quantification of atherosclerotic lesions of *ad libitum* (AL) or intermittent fasting (IF) males fed CD (**B**), females fed CD (**E**), males fed HFCD (**H**), and females fed HFCD (**K**). (**C**,**F**,**I**,**L**) Representative images of Oil Red O-stained sections of aortic valve of *ad libitum* (AL) or intermittent fasting (IF) males fed CD (**C**), females fed CD (**F**), males fed HFCD (**I**), and females fed HFCD (**L**). Arrows indicate the atherosclerotic lesions. Scale bars: 1 mm. Values are expressed as means ± SEM (*n* = 5–7/group); ns: not significant; ** *p* < 0.01, *** *p* < 0.001.

To examine the effect of the IF regimen on atherosclerosis development in *Apoe*^-/-^ mice, we determined the aortic sinus plaque sizes. Considering the distance from the heart to the aortic arch, the aortic root lesions were increased in both the AL and IF groups irrespective of sex and diet (Figure 8A,D,G,J). Interestingly, when mice were under CD, IF significantly decreased fatty streak lesions in the aortic root in both males and females as demonstrated by calculation of the AUC (Figure 8B,E) and evaluated by Oil Red O staining (Figure 8C,F). However, under HFCD, the lesion area was not modified by the IF regimen (Figure 8G–L). These data suggest that IF alleviates atherosclerosis progress in *Apoe*^-/-^ mice in a sex-independent manner when fed chow diet and that an atherogenic diet offsets this effect.

## 4. Discussion

Clinical parameters of cardiovascular diseases such as levels of circulating cholesterol and triglycerides have been reported to be reduced in animals and humans on every-other-day fasting, called intermittent fasting (IF). However, the majority of the results from these studies were conducted in healthy and overweight humans or in healthy or obese and diabetic mice that do not present cardiovascular abnormalities [38,39,40].

Only three studies to date have assessed the effect of IF in atherosclerosis-prone genetic mouse models [22,23,24]. However, discrepancies in the results regarding the weight gain reduction, the improvement of glucose metabolism, and the development of atherosclerosis were observed between these studies. Furthermore, only male mice were subjected to these interventions, and each study assessed IF in mice subjected to a single diet (chow diet, high-fat diet, or Western diet). 

Our study evaluated the relevance of IF in the improvement of glucolipid metabolism and atherosclerosis development in male and female *Apoe*^-/-^ mice fed either a chow diet (CD) or a high-fat and high-cholesterol atherogenic diet (HFCD). Our main findings were that the IF regimen prevented weight gain only in male *Apoe^-/-^* mice fed a chow diet, ameliorated glucose tolerance in a sex- and diet-independent manner, and reduced hypertriglyceridemia and atherogenesis only when *Apoe^-/-^* mice were fed a chow diet.

Despite the overall reduction in cumulative calorie intake in all groups submitted to IF compared to *ad libitum* ones, IF significantly reduced body weight gain and metabolic efficiency only in males fed CD, and it failed to reproduce the same effect in females fed CD or in mice fed HFCD. These findings suggest that the decrease in calorie intake alone cannot explain the observed decrease in body weight gain in males under CD. The sexual dimorphism regarding body weight benefits of IF in CD-fed *Apoe*^-/-^ mice was also described for wild-type C57BL/6J mice submitted to 10 h time-restricted feeding [41]. Moreover, IF by every-other-day fasting, the regimen we used in our study, has been reported to affect the level of sex hormones and gonadal function in rats, with increasing testosterone levels in males but not in females, and a significant change in gene expression in the gonads of males compared to females [42]. This could explain the beneficial effect of IF only in male mice fed CD. On the other hand, studies have shown that long-term feeding of a high-fat diet leads to similar cardiometabolic dysfunction in both male and female C57BL/6J mice [43], which is consistent with our finding that HFCD outweighs the beneficial IF effect, as both male and female *Apoe*^-/-^ mice fed HFCD were resistant to a reduction in body weight gain by IF. 

Metabolic efficiency is a hallmark of metabolic control of weight gain. While the bulk of energy intake is converted to heat by brown adipose tissue (BAT), metabolic efficiency constitutes a small part of the ingested energy that is stored as extra body energy deposits in white adipose tissue [32]. Interestingly, the effect of IF on weight loss was reported to be consequent to its effect on WAT browning and BAT activation [33]. 

We found that IF did not affect subcutaneous white adipose tissue (SC-WAT) weight irrespective of sex or diet. However, IF resulted in browning of WAT in mice fed chow diet independently of sex. This was accompanied by an increase in gene expression of browning markers and lipid metabolism enzymes. Deficiency in *Apoe* results in a smaller adipocyte size due to impairment of lipogenesis and a defect in lipid acquisition from the circulation. This renders *Apoe*^-/-^ mice resistant to adiposity when fed an obesogenic diet [34,44]. Accordingly, we found that HFCD feeding did not have a significant effect on body weight gain compared to CD feeding. Furthermore, HFCD by itself induced transformation of WAT towards a BAT-like phenotype, as previously reported [34,35]. This masked the effect of IF on WAT morphology when mice were fed HFCD. Interestingly, among the browning markers and lipid metabolism genes explored, only *Ucp1* gene expression was dramatically upregulated by IF in WAT when mice were fed HFCD. Intriguingly, brown adipocytes from mice fed CD acquired a white-like unilocular adipocyte phenotype, when submitted to IF in a sex-independent manner, accompanied by a significant decrease in *Ucp1* gene expression. However, BAT from mice fed HFCD presented an increase in genes induced by BAT activation. These results suggested that when mice were fed CD, IF induced a reduction in BAT activity that was offset by an increase in WAT browning. This is in line with the previously reported fasting suppression of *Ucp1* expression-induced thermogenesis in BAT [45,46,47] and the compensatory effect of white fat browning for defective BAT activity to maintain energy homeostasis metabolism [48]. However, when mice were fed HFCD, IF resulted in browning of WAT and activation of BAT. Interestingly, activated BAT and increased browning of WAT in *Apoe^-/-^* mice exacerbate atherosclerosis [49,50], and genetic deletion of *Ucp1* in *Apoe^-/-^* mice has been reported to prevent atherosclerotic plaque growth [49,50]. Furthermore, in humans, improvements in coronary heart disease risk factors by every-other-day fasting involved modulation of adipose tissue parameters [20].

IF effects on body fat remodeling were associated with an increase in glucose tolerance and improvement of insulin sensitivity [51,52,53]. We found an increase in glucose tolerance by IF in *Apoe^-/-^* mice irrespective of sex and diet. Unexpectedly, the improvement in glucose clearance was not a consequence of an enhancement in insulin sensitivity. Recent studies have demonstrated that glucose homeostasis could be maintained by non-insulin determinant pathways through a rearrangement of the gastrointestinal tract, glucose utilization in insulin-independent tissues/organs such as the brain and kidneys, or renal excretion of glucose, which is an insulin-independent process [54,55]. On the other hand, while glucose transporter GLUT4 is insulin-dependent and is responsible for the majority of glucose transport into muscle and adipose cells under anabolic conditions, GLUT1 is insulin-independent and is widely distributed in different tissues [56]. Although we did not explore these mechanisms, they could explain the IF-improved glucose tolerance in our model. 

We also found that IF decreased liver TG accumulation only in males fed chow diet. This was consistent with an increase in the expression of lipid catabolism genes in the liver. This effect was not observed in females fed CD. As the decrease in VLDL secretion was observed in both sexes, it could not explain the sexual dimorphism regarding liver TG content. This suggests that the decrease in TG content and VLDL secretion in males could be a result of increased liver lipid catabolism, while the absence of an IF effect in females could be a result of an equilibrium between lipid oxidation, synthesis, and secretion. 

There is a degree of discrepancy regarding the role of Apoe in hepatic lipid accumulation upon high-fat feeding. Karvia et al. showed that Apoe deficiency has a protective effect on diet-induced nonalcoholic fatty liver disease in mice through a mechanism involving a delay in post-prandial triglyceride (TG) clearance from their plasma [57]. However, Lu et al. demonstrated that Apoe deficiency promotes nonalcoholic fatty liver disease in mice by a mechanism involving a decrease in autophagy [58]. We found that IF exacerbated the mild HFCD-induced hepatic steatosis in a sex-independent manner in *Apoe^-/-^* mice. We found that this increase in liver TG content by IF was not associated with the regulation of lipolytic genes. However, the increased expression of Srebp-1c, which codes for a key transcription factor that regulates lipogenesis, could partially explain the exacerbation of hepatic steatosis, although there was no modification of the expression level of SREBP-1c target genes by IF. Interestingly, VLDL secretion was also inhibited by IF in *Apoe^-/-^* mice fed HFCD. These findings suggest that the high-fat diet outweighs the benefits of IF and worsens liver steatosis by disrupting the equilibrium between degradation, synthesis, and secretion of TG.

Although the levels of cholesterol content in the liver in all IF groups and the AL groups were comparable, we found that IF resulted in a decrease in gene expression of HMG-CoA synthase (*Hmgs*), which is an enzyme involved in cholesterol synthesis in males fed CD. Furthermore, in the same mice, IF significantly increased the expression of genes involved in cholesterol uptake by the liver, cholesterol efflux, and cholesterol bile secretion. These changes were not observed when mice were fed HFCD. These data suggest that when mice were fed CD, IF promoted an increase in cholesterol transport and excretion, while it induced a decrease in cholesterol synthesis.

Interestingly, while we did not observe any effect of IF on plasma cholesterol levels (total, HDL, and non-HDL cholesterol) in CD-fed mice, we found that cholesterol excretion into the bile, either as unesterified (free) cholesterol or after conversion to bile acids, was increased in these mice compared to their AL control. This is in line with the increase in the expression of genes involved in the biliary excretion of cholesterol, including *Abcg5/Abcg8* responsible for reducing circulating cholesterol. These data suggest that IF increases the clearance of cholesterol by reverse cholesterol transport (RCT), which is considered to be a main way for the body to excrete cholesterol [59]. We also found that the level of plasma TG was decreased by IF in CD-fed mice in a sex-independent manner. This could be explained by the observed decrease in hepatic VLDL-TG secretion. Although we did not measure lipoprotein lipase activity (LPL), the decrease in plasma TG concentration could also be attributed to an increase in LPL activity. Indeed, LPL, which is a key enzyme in lipid metabolism, hydrolyses triglycerides in circulating TG-rich lipoproteins and promotes the cellular uptake of chylomicron remnants, cholesterol-rich lipoproteins, and free fatty acids by adipose tissue and skeletal muscle [60]. Contrary to the beneficial effect of IF in mice fed CD, IF exacerbated both the plasma TG and cholesterol levels, which were increased by HFCD, and had no effect on biliary lipid secretion. We did not explore the molecular mechanisms of this effect, but we can hypothesize that IF in HFCD mice induced a decrease in LPL activity, a delay in post-prandial TG-rich lipoprotein clearance from the plasma, and/or an increase in intestinal lipid absorption.

Finally, and in keeping with the beneficial effects of IF on lipid metabolism in *Apoe*^-/-^ mice when fed CD, IF also reduced the development of aortic root sinus lesions in these mice in a sex-independent manner, but it failed to counteract atherosclerosis progression induced by HFCD. 

The effect of IF on atherosclerosis development has also been explored in the other frequently used model of mouse atherosclerosis, the low-density lipoprotein receptor-deficient (*Ldlr*^-/-^) model. The first study found that every-other-day fasting induced obesity and diabetes and exacerbated the development of spontaneous atherosclerosis in *Ldlr^-/-^* male mice fed a chow diet [24]. The difference with our results regarding glucose metabolism could be explained by the difference in genotypes, as LDL receptor but not apolipoprotein E deficiency increases diet-induced obesity and diabetes in mice [61]. Furthermore, aside from the fact that they only studied the effect of IF in *Ldlr*^-/-^ male mice, the results were obtained by IF of chow diet feeding. However, on a chow diet, *Ldlr*^-/-^ mice do not readily develop atherosclerosis, and thus a high-cholesterol diet with or without high fat is needed to provide the hyperlipidemic drive for atherogenesis in these mice [62]. Indeed, atherosclerosis development in *Ldlr^-/-^* mice was very slow when they were fed a chow diet for 3 months [63]. Therefore, under these conditions, *Ldlr^-/-^* mice could display significant individual differences in lesion development, which could bias the results of the IF regimen.

A more recent study showed that intermittent fasting cycles of 3 days of *ad libitum* feeding and 1 day of fasting ameliorated hypercholesterolemia, reduced atherosclerosis lesions, and increased plaque stability in *Ldlr^-/-^* male mice fed a high-fat diet [22]. Although we used a different mouse model and fasting cycles for IF intervention, our results with *Apoe*^-/-^ mice fed CD are comparable to the beneficial effect of IF on atherosclerosis development in *Ldlr^-/-^* male mice fed a high-fat diet. Taking into account that *Apoe^-/-^* mice exhibited higher plasma cholesterol and larger aortic root lesions with larger necrotic cores, and more smooth muscle cells and matrix at 3 months of atherogenic diet than did the *Ldlr*^-/-^ mice [62], one can suggest that the beneficial effect of IF depends on the stage of atherosclerosis development and the lesion area. Future studies will be needed to determine the plasma and aortic inflammatory profile as well as the plaque stability in our model.

It was recently reported that time-restricted feeding of a Western diet in *Apoe*^-/-^ male mice limits adiposity but fails to inhibit atherosclerosis progression and glucose intolerance [23]. Despite obtaining the same results regarding atherosclerosis development under HFCD, the effect of IF on glucose homeostasis and body weight gain is different. This discrepancy could be explained first by the difference in the fasting regimen treatment, as we used a more stringent intermittent fasting (every other day) compared to the 9 h per day used in the above study. The origin of fat in the diet could also be an explanation. Indeed, in our study, the dietary fat was derived from cocoa butter (diets rich in stearic acid), which could not have any effect on *Apoe^-/-^* body weight gain or glucose tolerance compared to chow diet [64]. However, the fat diet used in the study mentioned above is very obesogenic, as the body weight gain of *Apoe^-/-^* mice nearly reached 100% compared to their initial body weight after only 14 weeks of feeding [23]. This overweightness of mice was also accompanied by high fasting plasma glucose. However, diabetes hindered plaque regression in atherosclerotic mice, even after reduction of hyperlipidemia [65,66]. These results suggest that diabetes induced by an obesogenic diet is not suitable for assessment of the effect of IF on atherosclerosis development.

## 5. Conclusions

Our work assessed for the first time the same regimen of intermittent fasting (every other day) in both males and females on the development of both spontaneous atherosclerosis and atherogenic diet-induced progression of atherosclerosis in *Apoe*^-/-^ mice. Our results show that intermittent fasting of *Apoe*^-/-^ mice reduced body weight gain only in males fed chow diet, improved glycaemia in a sex and diet-independent manner, but reduced dyslipidemia and prevented atherosclerosis development irrespective of sex only when the mice were fed a chow diet.

Overall, the current work indicates that intermittent fasting could have potential clinical applications as an efficient therapy to improve glucose homeostasis and to treat atherosclerosis, but a degree of caution is warranted with regard to the dietary regimen and the overall eating patterns of the patients and/or stages of development of atherosclerosis. 

## Data Availability

Datasets generated in the present study are available from the corresponding author on reasonable request.

## Supplementary Materials

The following supporting information can be downloaded at: https://www.mdpi.com/article/10.3390/cells12040533/s1, Supplementary experimental procedure for hepatic secretion of VLDL-TG; Figure S1: Effect of intermittent fasting in *Apoe*^-/-^ mice on metabolic efficiency; Figure S2: Effect of intermittent fasting in *Apoe*^-/-^ mice on the plasma insulin level during glucose tolerance test; Figure S3: Effect of intermittent fasting in *Apoe*^-/-^ mice on white and brown adipose tissue weight. Figure S4: Effect of intermittent fasting in *Apoe*^-/-^ mice on hepatic very low-density-lipoprotein (VLDL)-TG secretion; Table S1: List of qPCR primer pair sequences and GeneBank accession numbers specific to each gene [67].
