# Supplementary material for: Intermittent Fasting Resolves Dyslipidemia and Atherogenesis in Apolipoprotein E-Deficient Mice in a Diet-Dependent Manner, Irrespective of Sex
Source: Cells. 2023 Feb 7;12(4):533. doi: 10.3390/cells12040533 (PMC9953823; doi:10.3390/cells12040533)
Supplement: Supplementary file 1 [file cells-12-00533-s001.zip › cells-2177876-supplementary.pdf]

# Supplementary Materials

## 1. Supplementary experimental procedures:

**Hepatic Secretion of very low-density lipoprotein (VLDL)-TG.** To estimate the rate of VLDL-TG secretion from the liver, mice were intraperitoneally injected with 500 mg/kg of tyloxapol (Sigma-Aldrich T0307), an inhibitor of lipoprotein lipase (LPL)[1]. Blood samples were collected from the tail vein 30 min before (time 0) and at 2, 3, and 4 h after injection. The TG concentration was determined with commercial colorimetric kits (Biolabo SAS, Maizy, France, #87656 #87319) based on the CHOD-PAP and GPO-PAP detection methods, coupling enzymatic reaction, and spectrophotometric detection of reaction end products (Dyasis, Grabels France). The TG production rate for individual mice was, therefore, calculated using the linear increment between the baseline value and 4 h and expressed as mg/dL.

1. Duerden, J.M.; Gibbons, G.F. Secretion and storage of newly synthesized hepatic triacylglycerol fatty acids in vivo in different nutritional states and diabetes. *Biochem. J.* **1988**, *255*, 929–935.

## 2. Supplementary Tables:

**Table S1**: List of qPCR primer pair sequences and GeneBank accession numbers specific to each gene.

| Gene symbol | Forward primer (5'-3') | Reverse primer (5'-3') | GeneBank |
|---|---|---|---|
| *Gapdh* | TGCACCACCAACTGCTTAGC | GGCATGGACTGTGGTCATGAG | NM_001289726 |
| *Pgc-1α* | AAAGGATGCGCTCTCGTTCA | GGAATATGGTGATCGGGAACA | NM_008904 |
| *Ucp1* | CCTGCCTCTCTCGGAAACAA | TGTAGGCTGCCCAATGAACA | NM_009463 |
| *Prdm16* | CAGCACGGTGAAGCCATTC | GCGTGCATCCGCTTGTG | NM_001291026 |
| *Cidea* | GCCGTGTTAAGGAATCTGCTG | TGCTCTTCTGTATCGCCCAGT | NM_007702 |
| *Cd36* | GCGACATGATTAATGGCACAGACG | TCCGAACACAGCGTAGATAGACC | NM_001159558.1 |
| *Acox1* | CTATGGGATCAGCCAGAAAG | AGTCAAAGGCATCCACCAAAG | NM_0015729.4 |
| *Cpt1-a* | CACCAACGGGCTCATCTTCT | CCTTCTATCGAATTTGCTCTGGTT | NM_013495 |
| *Acaca* | AGCAACATCACATCAGTCCTGT | CAGTGTAGCTGCATGACTATCTAGG | NM_133360.3 |
| *Elovl6* | TCTGATGAACAAGCGAGCCA | TGGTCATCAGAATGTACAGCATGT | NM_130450 |
| *Pparα* | CCCTGTTTGTGGCTGCTATAATTT | GGGAAGAGGAAGGTGTCATCTG | NM_011144 |
| *Ehhadh* | TTGGACCATACGGTTAGAGCC | GGATATCAGCACCTGCACAGA | NM_023737.3 |
| *Acaa1* | ACATCTCCGTGGGCAATGTT | CTCAGAAATTGGGCGATGCG | NM_001357516.1 |
| *Atgl* | TCCTTAGGAATGGCCTAC | TCCTCTTCCTGGGGGACAAC | NM_001163689.1 |
| *Hsl* | GATGCCATGTTGGCCAGAGAC | ACTTCTCGAAAGCCTTCTGGAACA | XM_030242180.1 |
| *Srebp1c* | CTGGCTTGGTGATGCTATGTTG | GACCATCAAGGCCCCTCAA | NM_001358315 |
| *Fas* | CGGCTGCGTGGCTATGATTATG | GCAGCTTGCCTTGTTCACCTTC | NM_007988 |
| *Scd1* | CGTGGGCGCGGTGAT | CAACACCATGGCGTTCCA | NM_009127.4 |
| *Elovl3* | GGACCTGATGCAACCCTATGA | TCCGCGTTCTCATGTAGGTCT | NM_007703 |
| *Dgat* | GGCGGTCCCCAACCAT | AGACAGGAGTGGAAAAACCAATAGA | NM_010046.3 |
| *Mttp* | TCAGGAAGCTGTGTCAGAATGAAG | TTTCAAGTCCTCCCAGGATCA | NM_008642 |
| *Srebp2* | CTGCAGCCTCAAGTGCAAAG | CAGTGTGCCATTGGCTGTCT | NM_033218 |
| *Hmgs1* | TGCATAGTAACACAGCAACAGAGC | TGCAGGGAGTCTTGGCACTTTC | NM_001291439.1 |
| *Hmgcr* | CCGGCAACAACAAGATCTGTG | ATGTACAGGATGGCGATGCA | NM_001360165 |
| *Scarb1/Srb1* | CGCCGACCCTGTGTTGTC | GGATGTCTAGGAACAAGGAATGCT | NM_001205083 |
| *Ldlr* | GCATCAGCTTGGACAAGGTGT | GGGAACAGCCACCATTGTTG | NM_010700 |
| *Vldlr* | TCCTGATTGCGAAGACGGTTCTG | ATGCGGCATGTTCTCATATGGC | NM_001347441.1 |
| *Abca1* | ATCGTGTCTCGCCTGTTCTCA | GTCCTTAATGCTGGTATCTCTTTGG | NM_013454 |
| *Cyp7A1* | CAGGGAGATGCTCTGTGTTCA | AGGCATACATCCCTTCCGTGA | NM_007824 |
| *Cyp27A1* | GCCTCACCTATGGGATCTTCA | TCAAAGCCTGACGCAGATG | NM_024264 |
| *Abcg5* | GGATCCAACACCTCTATGCTAAA | GGCAGGTTTTCTCGATGAACTG | NM_031884 |
| *Abcg8* | ATCCATTGGCCACCCTTGT | GCGTCTGTCGATGCTGGTC | NM_026180 |

## 3. Supplementary figures:

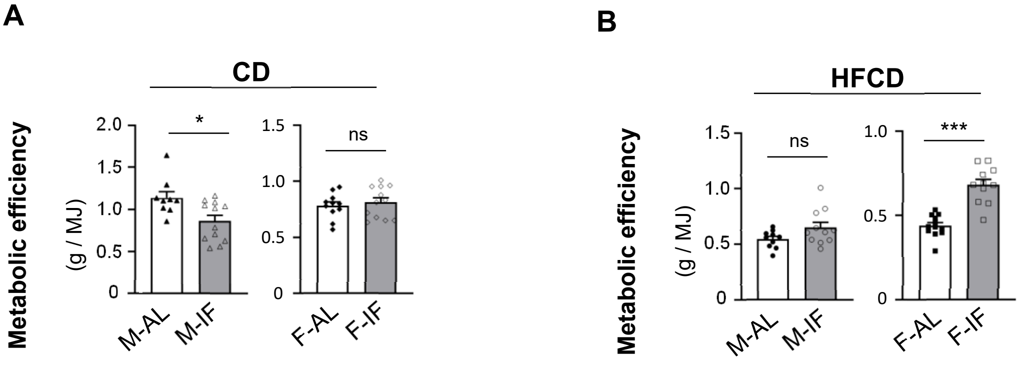


**Figure S1. Effect of intermittent fasting in *Apoe*$^{-/-}$ mice on metabolic efficiency.** (A–B) Metabolic efficiency was calculated as g of body weight gained per MJ of food consumed during the 16 weeks of intervention for *ad libitum* (AL) or intermittent fasting (IF) CD-fed mice (A) and HFCD-fed mice (B). Data are presented as means ± SEM (n = 12–14 per group); ns; not significant, *p < 0.05, **p < 0.01, ***p < 0.001.

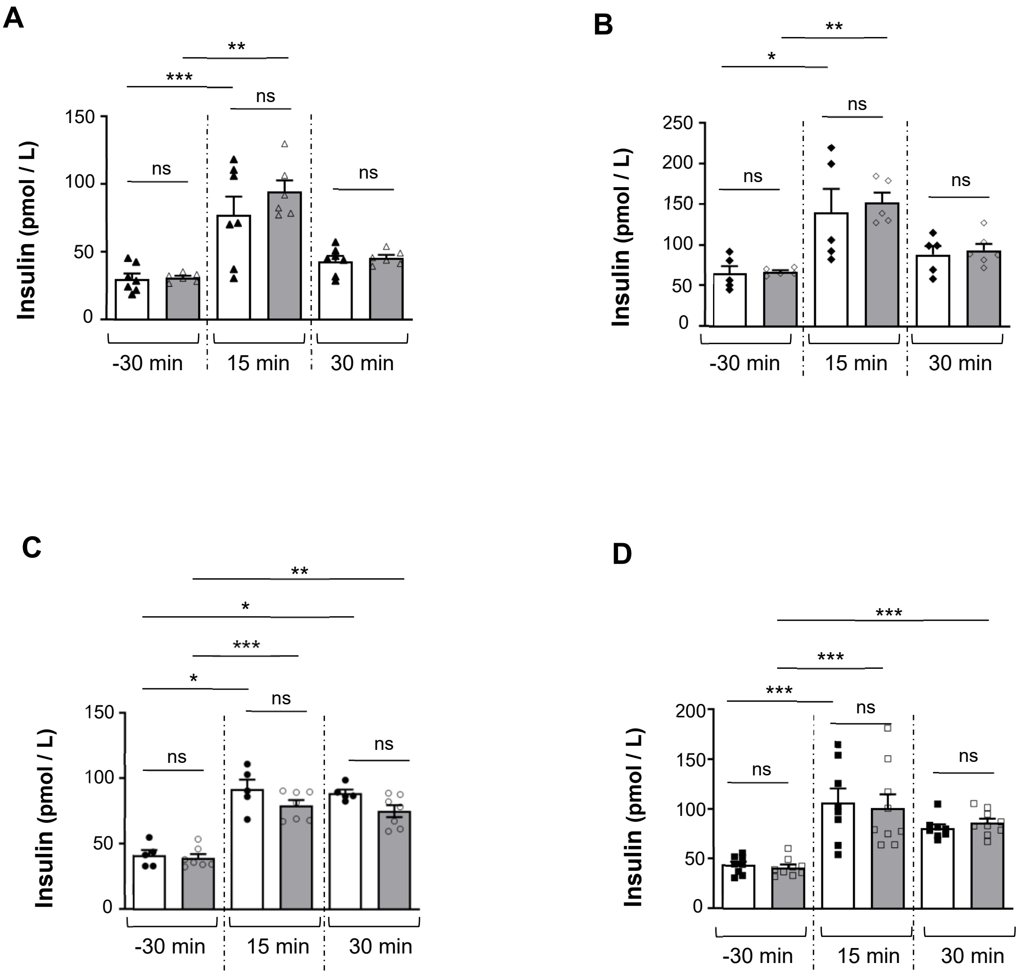


**Figure S2. Effect of intermittent fasting in *Apoe*$^{-/-}$ mice on the plasma insulin level during glucose tolerance test.** (A–D) Plasma insulin concentration at the indicated times after intraperitoneal bolus injection of glucose to fasted *ad libitum* (AL, white bars) or intermittent fasting (IF, grey bars) CD-fed males (A), CD-fed females (B), HFCD-fed males (C), and HFCD-fed females (D). Data are presented as means ± SEM (n = 5–9 per group); ns; not significant, *p < 0.05, **p < 0.01, ***p < 0.001.

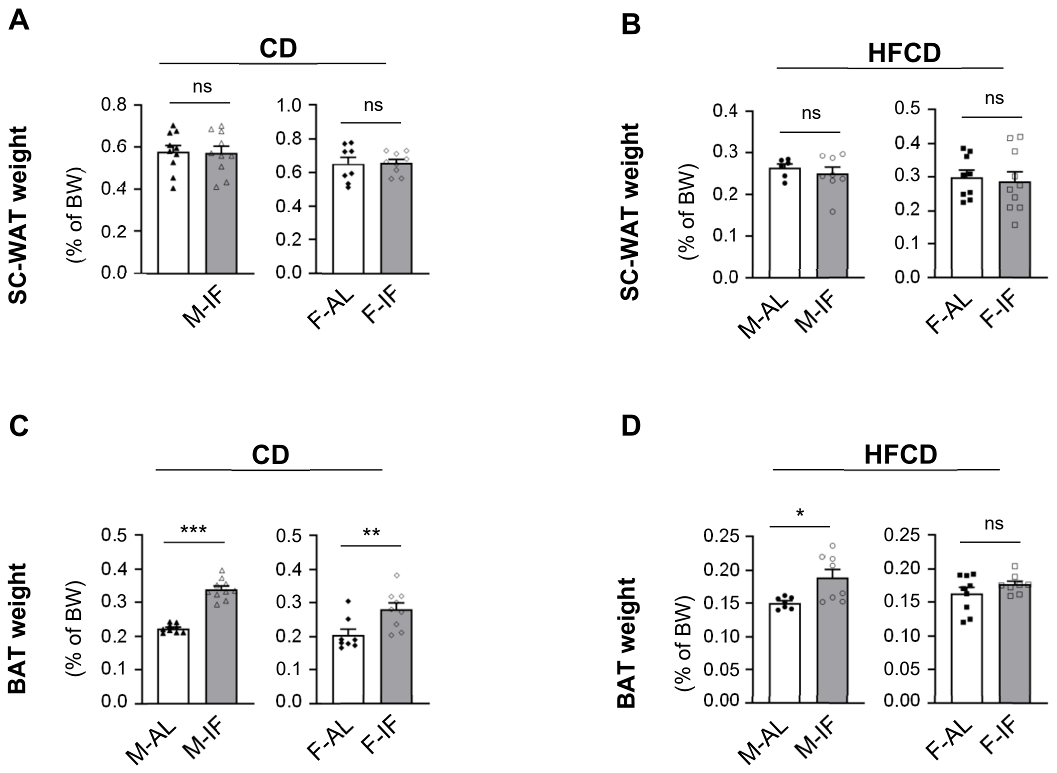


**Figure S3. Effect of intermittent fasting in *Apoe*$^{-/-}$ mice on white and brown adipose tissue weight.** (A,B) Subcutaneous white adipose tissue (SC-WAT) weights relative to body weight of *ad libitum* (AL) or intermittent fasting (IF) CD-fed mice (A) and HFCD-fed mice (B). (C,D) Brown adipose tissue (BAT), weights relative to body weight of *ad libitum* (AL) or intermittent fasting (IF) CD-fed mice (C) and HFCD-fed mice (D). Data are presented as means ± SEM (n = 6–10 per group); ns; not significant, $^{*}p < 0.05$, $^{**}p < 0.01$, $^{***}p < 0.001$.

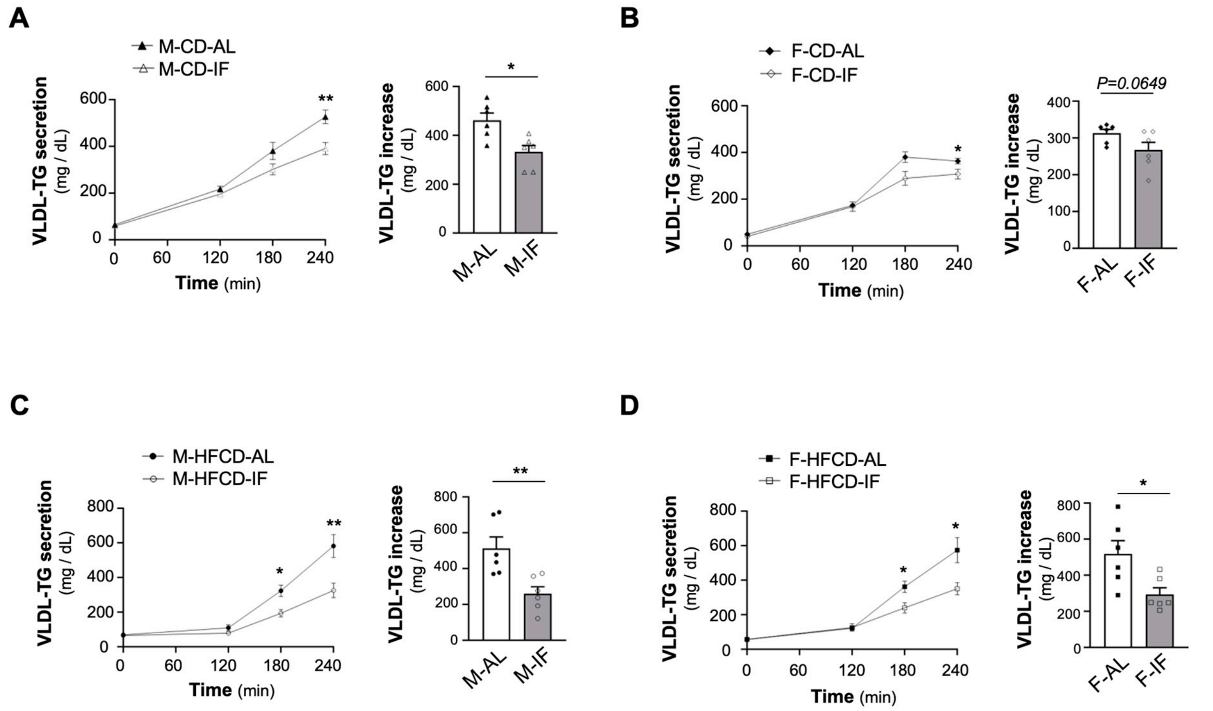


**Figure S4. Effect of intermittent fasting in *Apoe*$^{-/-}$ mice on hepatic very low-density-lipoprotein (VLDL)-TG secretion.** (A–D) Hepatic VLDL-TG secretion assessed at the indicated times following lipoprotein lipase inhibition by injecting 500 mg/kg of tyloxapol after a 4 h fast of *ad libitum* (AL) or intermittent fasting (IF) mice. Left panel: change in plasma VLDL-TG concentration for the indicated times, Right panel: rate of VLDL-TG secretion at 4 h. CD-fed males (A), CD-fed females (B), HFCD-fed males (C), HFCD-fed females (D). Data are means ± SEM (n = 6 per group).; ns; not significant, *p < 0.05, **p < 0.01, ***p < 0.001.
